# Effects of land use change on ecosystem services in freshwater wetlands in Bacalar, Mexico

**DOI:** 10.7717/peerj.18954

**Published:** 2025-03-13

**Authors:** Erika Betzabeth Palafox–Juárez, Mariana E. Callejas–Jiménez, Jorge A. Herrera–Silveira, Claudia Teutli–Hernández, Vera Camacho–Valdez, Jorge Omar López–Martínez

**Affiliations:** 1 Investigadores por México, Secretaría de Ciencia, Humanidades, Tecnologías e Innovación (SECIHTI), México, Mexico; 2 Departamento de Observación y Estudio de la Tierra, la Atmósfera y el Océano, El Colegio de la Frontera Sur, Chetumal, Quintana Roo, Mexico; 3 Centro de Investigación y de Estudios Avanzados del Instituto Politécnico Nacional, Merida, Yucatan, Mexico; 4 Escuela Nacional de Estudios Superiores, Mérida, Yuacatán, Universidad Nacional Autónoma de México; 5 Departamento de Conservación de la Biodiversidad, El Colegio de la Frontera Sur, San Cristobal de las Casas, Chiapas, Mexico; 6 Centro de Investigación en Ciencias de Información Geoespacial, Mérida, Yucatan, Mexico

**Keywords:** Land use change, Ecosystem services valuation, Inland wetlands, Mangroves, Bacalar Lagoon

## Abstract

Wetlands, such as those in Laguna Bacalar, Mexico, are highly productive and biodiverse ecosystems that provide a wide range of invaluable ecosystem services (ES). Despite their importance, these ecosystems are under significant threat from disturbances such as land-use changes, making them among the most endangered ecosystems worldwide. This study aimed to (1) assess the spatio-temporal variation of ecosystem services in Laguna Bacalar, Mexico, between 1999 and 2021 using medium-resolution satellite imagery from the Landsat sensor; and (2) estimate the monetary value of ES losses attributable to land-use changes by applying a unit value transfer method with global value coefficients based on data from Brander et al. (2024). Twenty-two key ESs were identified and associated with mangroves, inland wetlands, and the hydrological system. A total of 277 hectares of natural ecosystems were lost, leading to a reduction in the total value flow of ecosystem services (ES), which was estimated at 10,411,098 Int$/year over the study period. The loss of inland wetlands is particularly alarming due to their critical role in filtering agrochemicals and organic matter from the watershed. Increasing pressures from human activities, including urbanization and tourism, significantly contribute to the degradation of these ecosystems. This highlights the urgent need for responsible environmental management and the implementation of conservation strategies to protect their functionality and the invaluable ecosystem services they provide to local communities.

## Introduction

Ecosystem services (ES) are the features, functions, and processes of ecosystems that support and sustain human well-being (Costanza, 2020). They are defined as the direct and indirect benefits that people derive from ecosystems and are categorized into four main types: supporting, provisioning, regulating, and cultural services (MEA, 2005). In the context of the civilizational crisis and climate change, the valuation of ES has garnered significant attention in recent decades (Bateman et al., 2013) and their quantitative assessment serves as an effective tool for informing public policies aimed at ensuring their conservation (de Groot et al., 2012; Sannigrahi et al., 2019). The decline in the quality and quantity of ES is closely correlated with the type and frequency of anthropogenic activities, including deforestation, urbanization, and intensive agricultural practices, which disrupt ecosystem processes and diminish biodiversity (Costanza et al., 2014). Severely impacting ecosystem health and food production (Kovács-Hostyánszki et al., 2017; Porto et al., 2020), severely affecting ecosystem health and food production (Vanbergen & Insect Pollinators Initiative, 2013). Additionally, the reduction in vegetation cover has been shown to decrease carbon sequestration (Swangjang & Panishkan, 2021), increase erosion (Fernández-Díaz et al., 2022), and decrease biodiversity (Barbier, 2016; Shepard, Crain & Beck, 2011). These changes negatively impact the global economy and diminish the quality of life for human populations. Therefore, understanding the spatiotemporal variations of ES is crucial for identifying areas at greatest risk, prioritizing conservation efforts, and developing targeted public policies.

The concept of ecosystem services is inherently anthropocentric, with their value linked to the utility derived by humans from their consumption. In this context, it is important to distinguish between the total capacity an ecosystem service can provide, referred to as potential ecosystem services, and the portion of this capacity that is utilized, known as realized ecosystem services (Haines-Young & Potschin, 2010; Goldenberg et al., 2017). Studies that explicitly distinguish between potential and realized ecosystem services for mapping and valuation are limited in existing literature. In this context, our study focuses on potential ecosystem services, emphasizing the importance of conserving ecosystem processes beyond the realized services to ensure long-term benefits for humanity.

Wetlands are highly productive and biodiverse ecosystems, providing a wide range of ES including the regulation of biogeochemical cycles (*e.g*., carbon cycling), aquifer recharge, flood and drought regulation, and filtration of river runoff. Additionally, they serve as important sites for recreation and tourism (Gopal, 2013; Russi et al., 2013; Velázquez-Salazar et al., 2021). The ES provided by wetlands are valued at approximately USD 50.7 billion per year (Costanza et al., 2014); however, wetlands are fragile ecosystems that respond rapidly to disturbances, whether natural (*e.g*., hurricanes), or anthropogenic (*e.g*., land use change), which significantly affect their structure and function (Lomnicky, Herlihy & Kaufmann, 2019) and increasing their vulnerability (Bhowmik, 2019). Globally, wetlands are among the most threatened ecosystems, with an estimated 87% having undergone land-use changes or been dredged or filled to support agriculture, urban expansion, and tourism (Clarkson, Ausseil & Gerbeaux, 2013; Davidson, 2014; Yan et al., 2023).

The situation of wetlands in Mexico is worrying. Despite being one of the countries with the largest wetland coverage globally—accounting for 6.7% of the world’s mangrove coverage—and having robust conservation legislation, these ecosystems continue to be degraded by anthropogenic activities (Clarkson, Ausseil & Gerbeaux, 2013; Davidson & Finlayson, 2019; Russi et al., 2013). The state of Quintana Roo, located in the Mexican Caribbean, is one of the world’s leading tourist destinations. Since the 1970s, this status has driven the development of large-scale infrastructure projects, such as Cancun and the Riviera Maya (Espinosa Coria, 2013; Gómez Pech, Barrasa García & García de Fuentes, 2018). Tourism driven by Quintana Roo’s natural attractions is among the most economically impactful activities in Mexico, contributing 23% to the national tourism gross domestic product (GDP) in 2022 (INEGI, 2023). However, the transformation of natural ecosystems through land use change has resulted in the decline and loss of ES associated with coastal dunes, wetlands, forests, and biodiversity (Ellis, Romero Montero & Hernández Gómez, 2017; Huchin Ochoa et al., 2022; Teutli-Hernández et al., 2024). In 2006, a campaign was initiated to promote tourism in southern Quintana Roo, centered around Bacalar Lagoon, also known as the ‘*Lagoon of the Seven Colours’* (Gómez Pech, Barrasa García & García de Fuentes, 2018; Secretary of Tourism of the Government of Mexico (SECTUR), 2014). However, it was not until 2015, with the massive arrival of sargassum to the coasts of Quintana Roo (Uribe-Martínez et al., 2022), that Laguna Bacalar emerged as a viable option to diversify tourism and attract investment in the region. This development resulted in a remarkable surge in visitor numbers, increasing by up to 750% compared to previous years (INEGI, 2023). This surge in visitor numbers led to the development of infrastructure to meet the growing demand for tourism services and support the local population, jeopardizing the natural landscapes and the ecological integrity of Bacalar Lagoon (Gómez Pech, Barrasa García & García de Fuentes, 2018; Secretary of Tourism of the Government of Mexico (SECTUR), 2005, 2014).

Bacalar Lagoon, renowned for its vibrant colors, experienced a mesotrophic process in the mid-2020s due to excessive nutrients and pollutants inputs from adjacent agricultural areas, triggered by an extreme rainfall event (Álvarez-Legorreta, 2025). These impacts have far-reaching implications, including a significant mortality event in the populations of *Pomacea flagellata snail* (De Jesús-Navarrete et al., 2019; De Jesús-Navarrete, 2025). Additionally, the loss of Bacalar Lagoon’s distinctive colors could adversely affect tourism activities, thereby impacting the region’s economy (Torrescano-Valle et al., 2025). This suggests that processes occurring in the upper watershed, such as land use change for agriculture and deforestation (Ellis et al., 2020; Huchin Ochoa et al., 2022), as well as the development of urban and tourism infrastructure along the lagoon coast (Palafox-Juárez et al., 2022) significantly impact lagoon ecosystems, highlighting their high connectivity and fragility. Economic valuation studies for the region are scarce and primarily focus on the cultural ES perceived in ecosystems of tourist interest. Notable examples include the reefs of the Mesoamerican Reef System (Ruiz de Gauna et al., 2021), mangroves and reefs in the north of the state (Cortés-Gómez, Cervantes-Martínez & Arce-Ibarra, 2023; Sánchez-Quinto et al., 2020) Mayan jungle landscape (Alpuche-Álvarez et al., 2019) and sinkholes (May-Arias, Arroyo Arcos & Hernández Aguilar, 2024). However, no estimates of ES valuation exist for the Bacalar region, despite their critical importance for developing management and conservation strategies for fragile ecosystems like the wetlands of Laguna Bacalar.

In recent decades, advancements in satellite information accessibility and computational resources have positioned remote sensing as a powerful tool for assessing ES (Davidson & Finlayson, 2018; Long et al., 2022). Currently, a wealth of historical satellite data is available at different spatial, spectral, and temporal resolutions, enabling the mapping and monitoring of ES supply and demand (Burkhard et al., 2009; Fu et al., 2014; Long et al., 2022), especially in regions with limited funding for ES evaluation (Johnston & Wainger, 2015). In this context, various approaches have been developed to assign monetary values to ES using satellite imagery, further supporting the argument that biodiversity conservation generates significant benefits (Brander, 2013; Richardson et al., 2015; Wong et al., 2015). In this case, remote sensing combined with the benefit transfer method, also known as the value transfer method, has been extensively employed to assess ES across various locations and scales. This facilitates the transfer of results from existing valuation studies to other areas with comparable ecosystems and beneficiaries, significantly reducing both time and costs (Plummer, 2009; Mendoza-González et al., 2012; Wong et al., 2015; Fernández-Díaz et al., 2022).

In this context, this study aimed to: (1) compare the spatiotemporal variation of potential ecosystem services in Laguna Bacalar, Mexico, for 2 years (1999 and 2021), using medium-resolution satellite imagery from the Landsat sensor; and (2) estimate the economic value of ecosystem services lost due to land-use changes through a unit value transfer analysis, incorporating The Economics of Ecosystems & Biodiversity (2010) methodologies and the monetary estimates proposed by Brander et al. (2024).

## Materials and Methods

### Study area

The “Bahía de Chetumal y otras” watershed covered an area of 757,465 hectares and extended across the states of Campeche and Quintana Roo. This exoreic basin, with elevations ranging from 0 to 190 m above sea level (INEGI, 2010), is characterized by Rendzina, Litosol, and Gleysol soil, which rested on a limestone plateau composed of calcite, dolomite, and gypsum. The highly permeable subsurface facilitated substantial groundwater flow, which supported the formation of cenotes and freshwater lakes (Bautista et al., 2011).

The predominant vegetation consisted of medium and low tropical forests, grasslands, and agricultural lands. However, land-use changes driven by urbanization, agriculture, and livestock farming resulted in significant ecosystem loss. In the municipality of Bacalar alone, an estimated 76,000 hectares of forests and wetlands were lost over the past 27 years, primarily due to agricultural, livestock, and tourism expansion (Miranda, 1958; Comisión Nacional Forestal (CONAFOR), 2018; INEGI, 2023; Huchin Ochoa et al., 2022).

The lagoon system in southern Quintana Roo originated from a series of geological fractures that shaped the region’s hydrology. This system included the Río Hondo, which forms the border with Belize, and six interconnected freshwater lagoons: San Felipe, Guerrero, Chile Verde, Milagros, Bacalar, and the Bahía de Chetumal. These water bodies are interconnected through surface and subterranean flows *via* channels and runoff, ultimately discharging into the Caribbean Sea (Rebolledo-Vieyra, 2009; Hernández-Arana et al., 2015; Torrescano-Valle et al., 2025).

**Figure 1:**
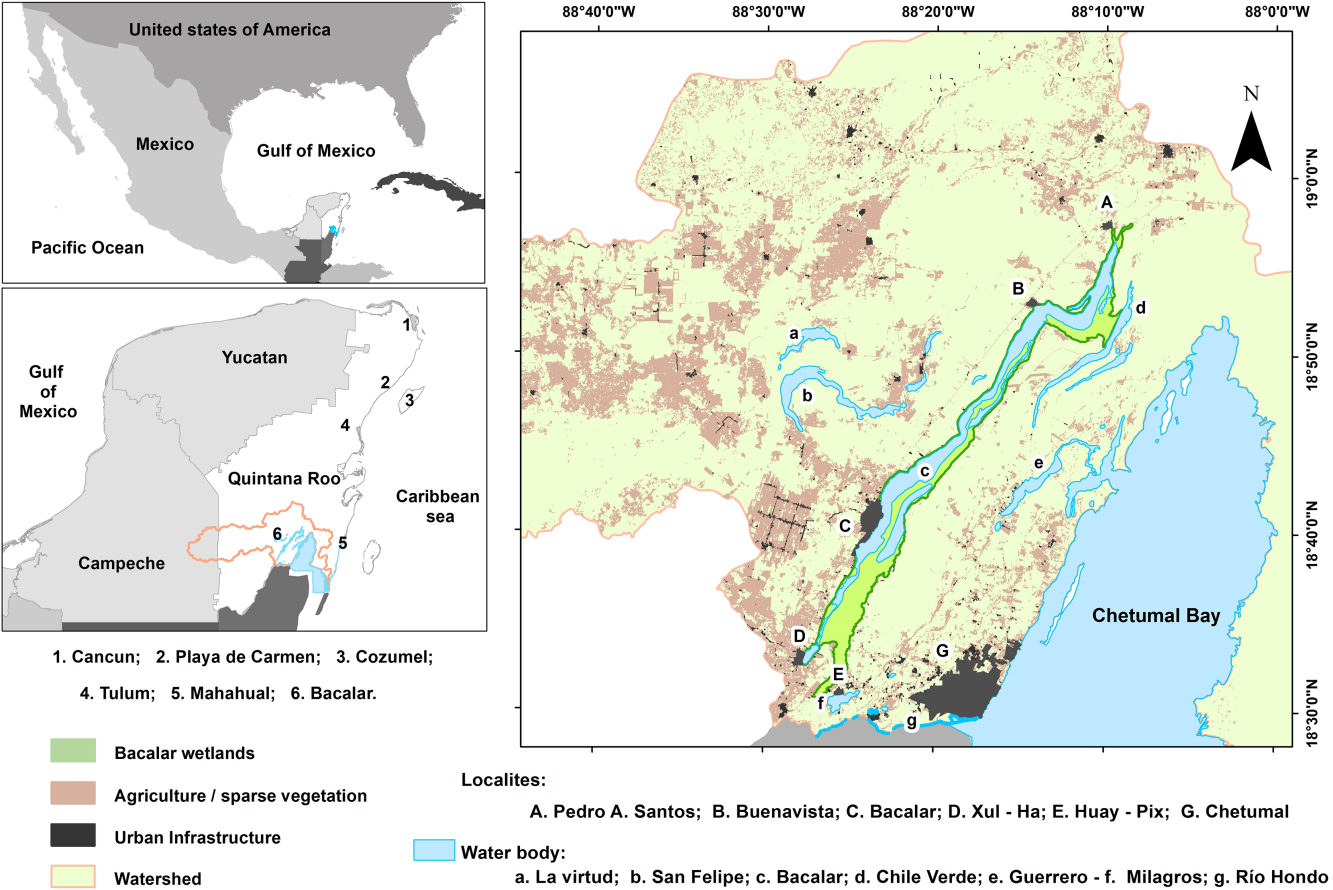
Study area location. Numbers represent the leading tourist destination on Quintana Roo. The distribution of the Bacalar wetlands is shown in green. Capital letters correspond to the main human settlements, and lowercase letters list the water bodies associated with the Bacalar Lagoon.

Bacalar Lagoon, situated in the central part of this watershed, is the largest freshwater body of the Yucatan Peninsula and hosts one of the world’s most significant regions of stromatolites (microbial reefs formed by cyanobacterial activity) (Yanez-Montalvo et al., 2020). Additionally, several types of wetlands are distributed along its coastline (Hernández-Arana et al., 2015; Palafox-Juárez et al., 2022). The lagoon featured a main body of water, interconnected channels, and dolines. It was characterized by a narrow, elongated shape, extending to 53 km in length, with a variable width ranging from 20 m and 2 km, and an average depth of the lagoon was 9 m, with dolines reaching a maximum depth of 60 m (Carrillo et al., 2024) (Fig. 1).

### Characterization of the ecosystems of Bacalar Lagoon

The ecosystems of Laguna Bacalar, as detailed by Palafox-Juárez et al. (2022) were classified categorized following the approach of The Economics of Ecosystems & Biodiversity (2010) and Brander et al. (2024), which are described as: (1) Mangrove vegetation in flood zones with silty-sandy soil enriched with organic material residues. The dominant vegetation consists of dwarf red mangrove (*Rhizophora mangle*), accompanied by smaller proportion of species such as *Conocarpus erectus*, *Morella cerifera*, and *Bonellia macrocarpa* sp., as well as *Carex* sp. (2) Inland wetlands, mainly distributed along the lagoon’s western shore, are characterized by rocky soil with slopes of lees than <45°, and surface water inputs. The characteristic vegetation includes species such as *Bucida buceras*, *Ficus* sp., *Coccoloba uvifera*, *R. mangle*, *C. erectus*, *Metopium brownei*, *Cordia dodecandra*, *Lysiloma latisiliquum*, *Thrinax radiata*, and *Acoelorraphe wrightii*. (3) Water (Lakes and rivers) forms the matrix that connects the surrounding ecosystems through complex hydrodynamic processes.

### Image acquisition and pre-processing

Ortho-rectified images were acquired from Landsat 5 TM and 9 OLI-2; TIRS-2 sensors (USGS; https://earthexplorer.usgs.gov) (Table 1). The spectral and spatial resolution of the images varied between sensors. Landsat 5 TM provided seven bands with a spatial resolution of 30 m, including one thermal band with 120 m resolution, whereas Landsat 9 offered bands with 30 m spatial resolution (OLI-2), two thermal bands with 100 m (TIRS-2), and one panchromatic band with 15 m resolution (Table 2). Images from February 1999 and October 2021 were selected due to their minimal cloudy cover and the presence of sufficiently green vegetation.

**Table 1:** List of landsat images used to carry out the analysis.

ID	Image name	Path	Row	Year	Month	Day
1	LT05_L2SP_019047_19990222_20240112_02_T1	19	47	1999	2	22
2	LC09_L2SP_019047_20211031_20230507_02_T1	19	47	2021	10	31

**Table 2:** Characteristics of landsat sensors (https://www.usgs.gov/landsat-missions/landsat-satellite-missions).

Sensor	Band	Name	Resolution (m)	Wavelength (nm)
Landsat 5 TM	B01	Blue	30	0.45–0.52
	B02	Green	30	520–600
	B03	Red	30	630–690
	B04	NIR	30	760–900
	B05	SWIR 1	30	1,550–1,750
	B06	Thermal	120	10.40–12.50
	B07	Mid-Infrared	30	2.80–2.35
Landsat 9 OLI-2; TIRS-2	B01	Costal/Aerosol	30	433–453
	B02	Blue	30	450–515
	B03	Green	30	525–600
	B04	Red	30	630–680
	B05	NIR	30	845–885
	B06	SWIR 1	30	1,560–1,660
	B07	SWIR 2	30	2,100–2,300
	B08	PAN	15	500–680
	B09	Cirrus	30	1,360–1,390
	B10	TIRS 1	100	10,300–11,300
	B11	TIRS 2	100	11,500–12,500

Supervised classification was conducted using the blue, green, red, NIR, and SWIR 1 bands from Landsat 5, as well as the SWIR 2 band from Landsat 9. All selected bands had a uniform spatial resolution. To mitigate atmospheric scattering effects, each image underwent radiometric and atmospheric correction following the methodology of Chuvieco & Huete (2009), using the Fast Line of Sight Atmospheric Analysis of Spectral Hypercubes (FLAASH) in ENVI 5.4 software (Harris Geospatial Solutions, 2016).

### Data field of regions of interest and supervised classification of Bacalar Lagoon

Based on the ecosystems identified in Laguna Bacalar, regions of interest (ROIs) were delineated using the following approaches: (i) field visits, supplemented by the empirical knowledge of local inhabitants to identify inland wetland zones, and (ii) analysis of imagery from the Google Earth satellite viewer. Finally, a total of 312 regions of interest (ROIs) were established and distributed as follows: mangrove vegetation (139), inland wetlands (49), and water (91). Additionally, to assess the expansion of human activities, ROIs were assigned to a fourth category termed ‘human-modified’ (34). Each training site was georeferenced using a Garmin 62S global positioning system (GPS) with a positional error of approximately 1 m. Supervised classifications were conducted on Landsat 5 TM (1999) and Landsat 9 OLI-2/TIRS-2 (2021) images using the maximum likelihood algorithm, which assigns pixels to classes based on the highest probability of association (Chuvieco & Huete, 2009; Taylor, Kumar & Reid, 2011). The analyses were performed in R software (R Core Team, 2021).

To assess map uncertainty, a cross-validation was conducted following the methodology proposed by Olofsson et al. (2014). This involved utilizing a high-resolution Sentinel-2 image and generating 140 points through stratified random sampling

### Monetary estimation of the ecosystem services of Laguna Bacalar

To estimate the changes in the value of ES provided by Bacalar’s ecosystems from 1999 and 2021, a Unit Value Transfer analysis was conducted, using Eq. (1) (Troy & Wilson, 2006). The monetary estimates proposed by Brander et al. (2024) for mangroves, inland wetlands, and lakes and rivers, were employed as baseline reference values. These values were subsequently multiplied by the surface area (ha) of each ecosystem type identified in Laguna Bacalar for 1999 and 2021.

(1) $$V\left( {E{S_i}} \right) = \; \mathop \sum \limits_{k = 1}^n A\left( {L{U_i}} \right)*V\left( {E{S_{ki}}} \right)$$

where A(LUi) = area of wetland type (i) and V(ESki) = annual value per unit area for the type of ecosystem service (k) generated by wetland type (i).

## Results

### Image classification and validation of cover class

**Figure 2:**
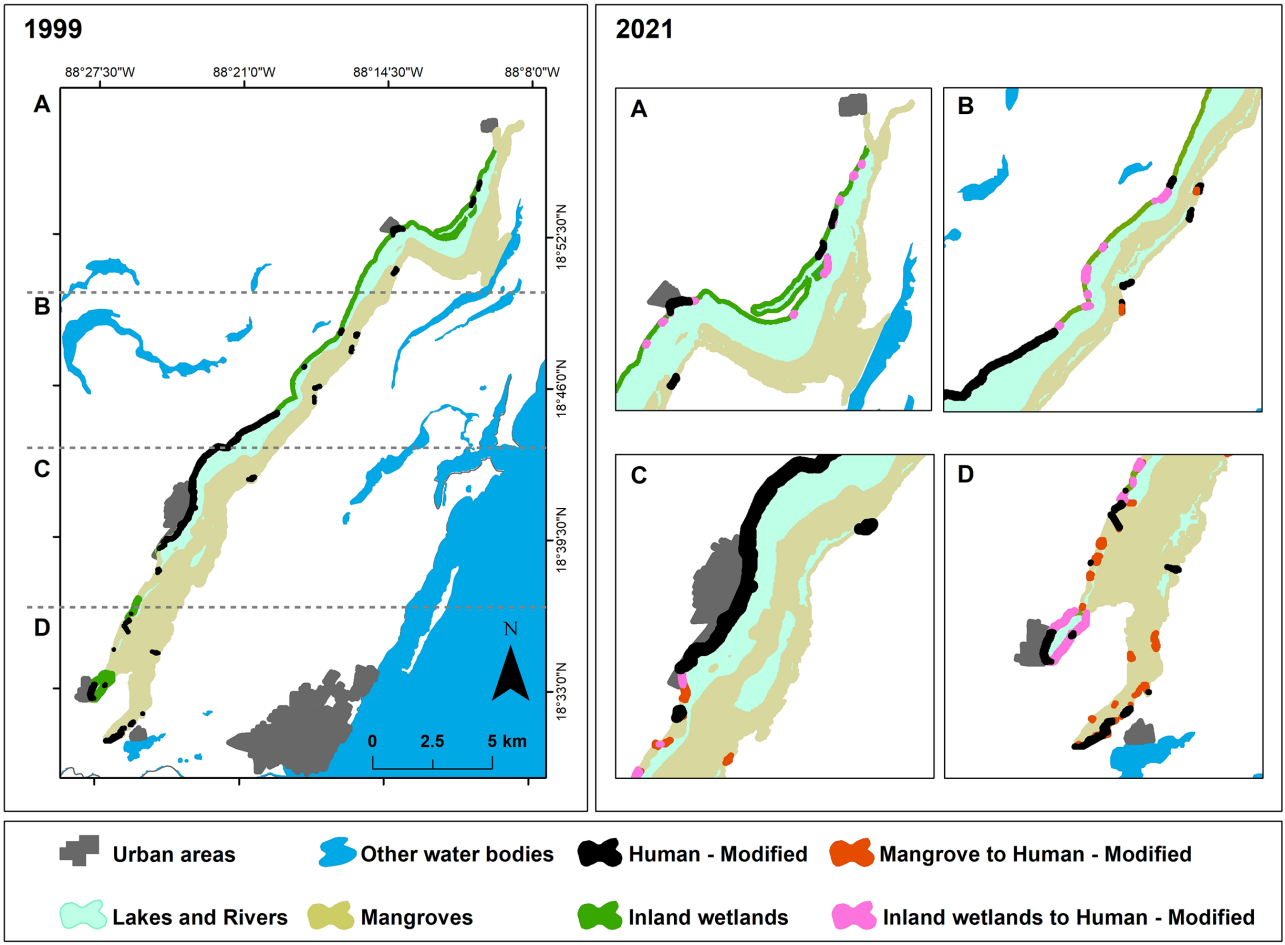
Cover of mangroves, Inland wetlands, and Lakes & Rivers in Bacalar from 1999 and 2021.

Figure 2 illustrates the spatial distribution of the ecosystems analyzed, along with the human-modified class, for 1999 and 2021. The ecosystems with the highest spatial representation in Laguna Bacalar were observed to be the mangrove and water ecosystems, collectively accounting for approximately 95% of the total area studied, followed by inland wetlands and the human-modified class (Table 3). The supervised classification achieved an overall map accuracy of 92.2% when compared to the reference data. Furthermore, the ecosystem-specific validation based on the methodology of Olofsson et al. (2014) indicated that the mangrove ecosystem was the most accurately classified, whereas the human-modified class exhibited the lowest classification accuracy (Table 4).

**Table 3:** Overall, user and producer accuracies standard error in percentages per cover class.

Land use/Land cover	User’s accuracy	Producer’s accuracy	Overall’s accuracy
Lakes & rivers	86.11 (±0.0576)	88.57 (±0.0538)	92.186 (±0.0227)
Mangrove	91.66 (±0.0461)	94.28 (±0.0392)	
Inland wetlands	80 (±0.0676)	80 (±0.0676)	
Human-Modified	78.787 (±0.0712)	74.28 (±0.0739)	

**Table 4:** Area (ha) of each land cover and land use for 1999 and 2021.

Land cover/Land use	1999		2021		Change	
	ha	%	ha	%	ha	%
Lakes & rivers	7,474.1	50.2	7,469.1	50.2	−5.0	−0.0
Mangroves	6,720.7	45.1	6,690.3	44.9	−29.4	−0.2
Inland wetlands	546.2	3.7	303.8	2.0	−242.4	−1.6
Human-Modified	147.7	1.0	424.6	2.9	276.9	1.9
Total	14,887.7	100	1487.7	100		

### Land cover changes (1999–2021)

From 1999 and 2021, 29 ha of mangrove and 242 ha of inland wetlands were lost, primarily corresponding to an increase of 277 ha modified for urban and tourist infrastructure along the west coast. Additionally, there was a reduction of 5 ha in lakes and rivers (Fig. 2).

### Analysis of the spatiotemporal variation of ecosystem services

**Figure 3:**
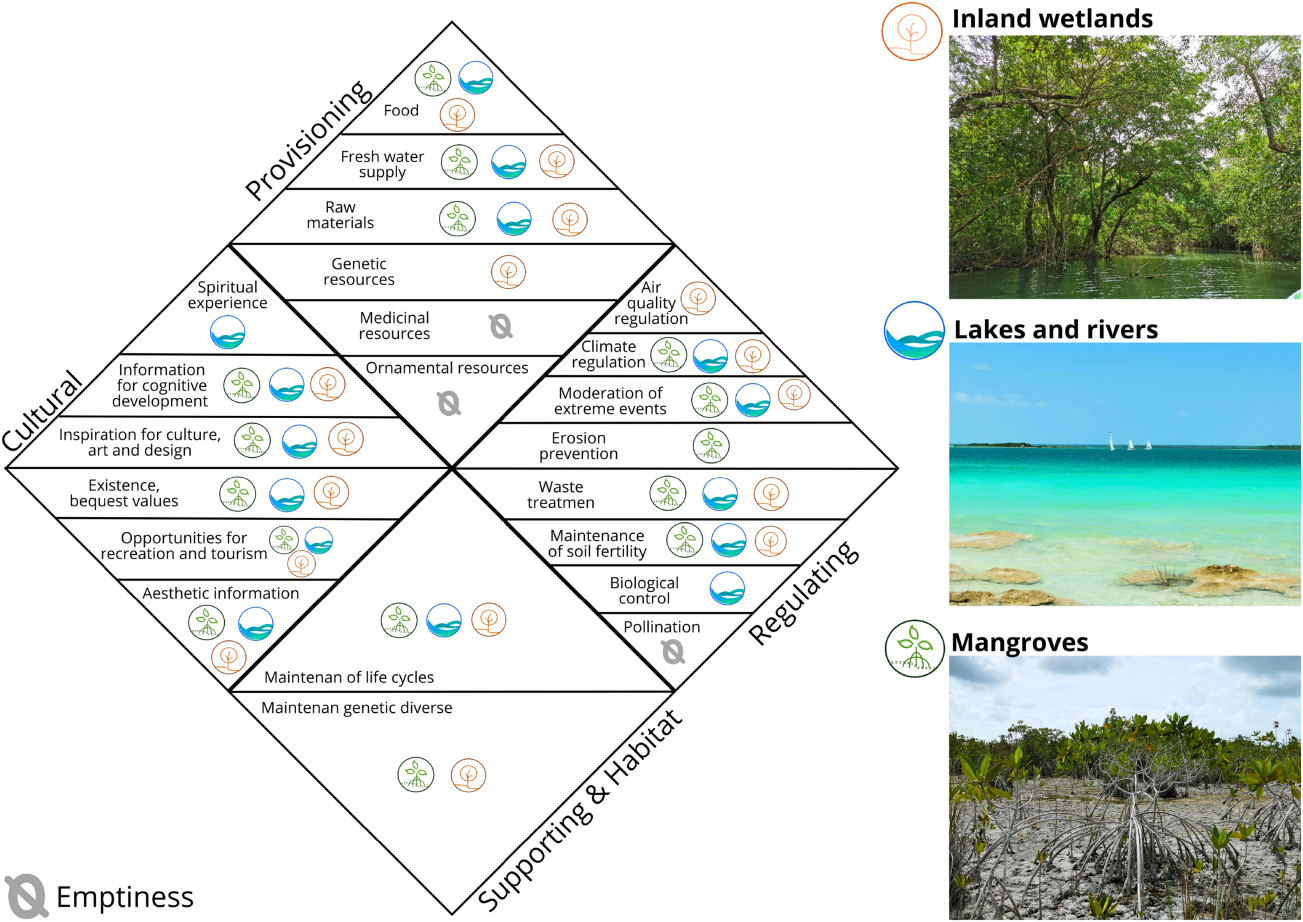
ES provided by Bacalar ecosystems based on The Economics of Ecosystems & Biodiversity (2010) and Brander et al. (2024).

**Figure 4:**
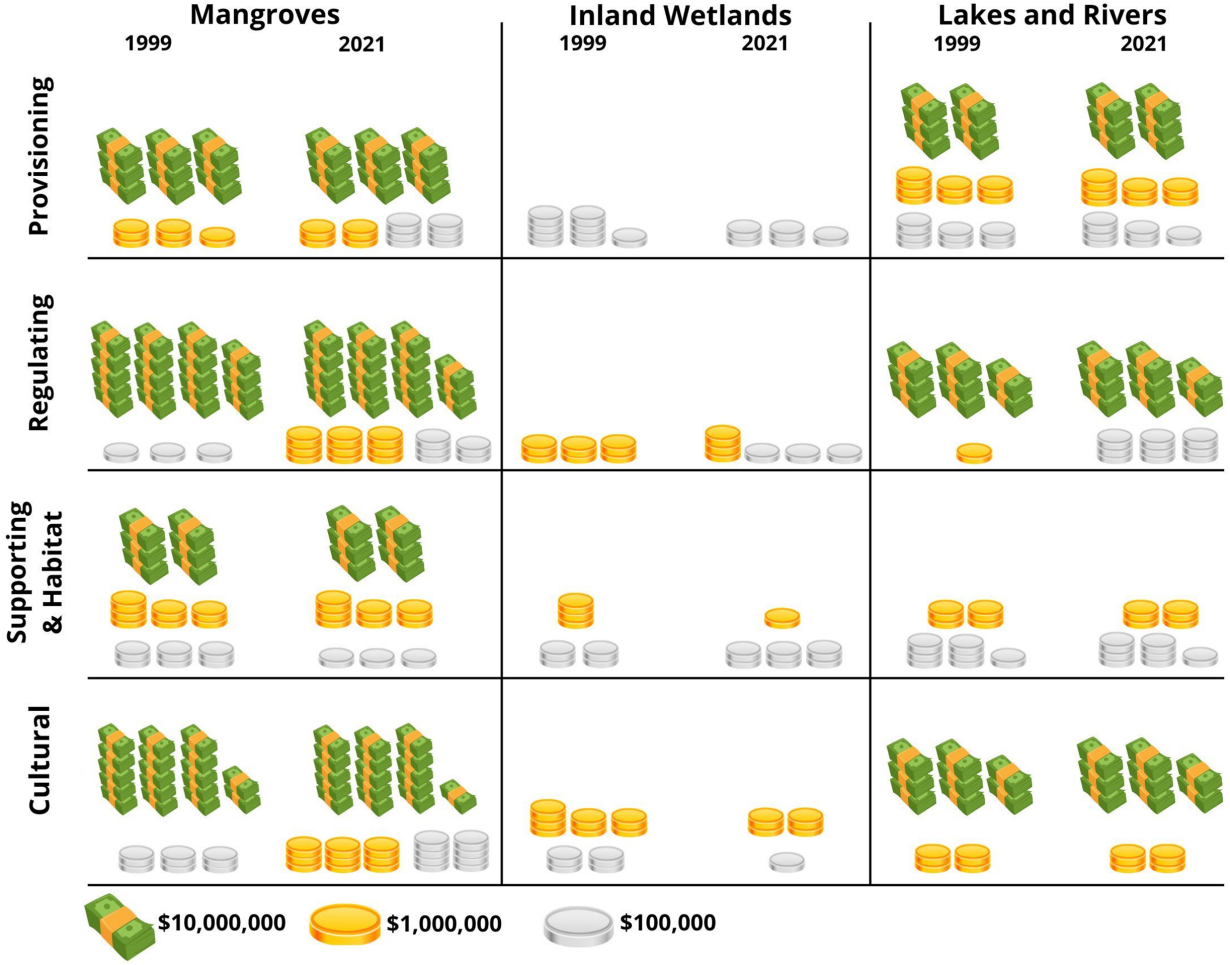
Changes in the value of ecosystem services provided by mangroves, inland wetlands, and lakes & rivers of Bacalar. Values reported in Int$/year.

Of the 22 ecosystem services provided by ecosystems globally (The Economics of Ecosystems & Biodiversity, 2010; Brander et al., 2024), the mangroves, inland wetlands, and lakes and rivers in Bacalar provide four provisioning, seven regulating, two supporting, and six cultural ES (Fig. 3). Based on the results obtained, from 1999 to 2021, 277 ha of natural ecosystems were modified, resulting in an overall decrease in the value of ecosystem services amounting to 10,411,098 Int$/year (Fig. 4, Tables 5–7).

**Table 5:** Changes in the value of ecosystem services of the inland wetlands of Bacalar from 1999 and 2021.

Inland wetlands ecosystem services	Reference values (int$ 2020/ha/year)	Estimated to Bacalar		Chance
		1999	2021	−242 ha/$
		546 ha	304 ha	
Provision				
Food	$612	$334,152	$186,048	−$148,104
Water	$873	$476,658	$265,392	−$211,266
Raw materials	$18	$9,828	$5,472	−$4,356
Genetic resources	$229	$125,034	$69,616	−$55,418
Subtotal		$945,672	$526,528	−$419,144
Regulation				
Air quality regulation	$2,485	$1,356,810	$755,440	−$601,370
Climate regulation	$185	$101,010	$56,240	−$44,770
Moderation of extreme events	$4,969	$2,713,074	$1,510,576	−$1,202,498
Waste treatment	$2,603	$1,421,238	$791,312	−$629,926
Maintenance of soil fertility	$812	$443,352	$246,848	−$196,504
Subtotal		$6,035,484	$3,360,416	−$2,675,068
Support				
Maintenance of life cycles	$4,759	$2,598,414	$1,446,736	−$1,151,678
Maintenance genetic diverse	$1,483	$809,718	$450,832	−$358,886
Subtotal		$3,408,132	$1,897,568	−$1,510,564
Cultural				
Aesthetic information	$493	$269,178	$149,872	−$119,306
Opportunities for recreation and tourism	$12,899	$7,042,854	$3,921,296	−$3,121,558
Existence, bequest values	$63	$34,398	$19,152	−$15,246
Inspiration for culture, art, and design	$101	$55,146	$30,704	−$24,442
Information for cognitive development	$120	$65,520	$36,480	−$29,040
Subtotal		$7,467,096	$4,157,504	−$3,309,592
Total		$17,856,384	$9,942,016	−$7,914,368

**Table 6:** Changes in the value of ecosystem services of the mangroves of Bacalar from 1999 and 2021.

Mangroves ecosystem services	Reference values (int$ 2020/ha/year)	Estimated to Bacalar		Chance
		1999	2021	−29 ha/$
		6,720 ha	6,690 ha	
Provision				
Food	$6,791	$45,635,520	$45,431,790	−$203,730
Water	$1,623	$10,906,560	$10,857,870	−$48,690
Raw materials	$5,729	$38,498,880	$38,327,010	−$171,870
Subtotal		$95,040,960	$94,616,670	−$424,290
Regulation				
Air quality regulation	$1,323	$8,890,560	$8,850,870	−$39,690
Climate regulation	$1,375	$9,240,000	$9,198,750	−$41,250
Moderation of extreme events	$14,388	$96,687,360	$96,255,720	−$431,640
Waste treatment	$3,189	$21,430,080	$21,334,410	−$95,670
Erosion prevention	$7,030	$47,241,600	$47,030,700	−$210,900
Maintenance of soil fertility	$1,028	$6,908,160	$6,877,320	−$30,840
Subtotal		$190,397,760	$189,547,770	−$849,990
Support				
Maintenance of life cycles	$4,078	$27,404,160	$27,281,820	−$122,340
Maintenance genetic diverse	$5,982	$40,199,040	$40,019,580	−$179,460
Subtotal		$67,603,200	$67,301,400	−$301,800
Cultural				
Aesthetic information	$334	$2,244,480	$2,234,460	−$10,020
Opportunities for recreation and tourism	$6,118	$41,112,960	$40,929,420	−$183,540
Existence, bequest values	$14,299	$96,089,280	$95,660,310	−$428,970
Inspiration for culture, art, and design	$3,890	$26,140,800	$26,024,100	−$116,700
Information for cognitive development	$749	$5,033,280	$5,010,810	−$22,470
Subtotal		$170,620,800	$169,859,100	−$761,700
Total		$523,662,720	$521,324,940	−$2,337,780

**Table 7:** Changes in the value of ecosystem services of the Lakes & rivers of Bacalar from 1999 and 2021.

Lakes & rivers ecosystem services	References values (int$ 2020/ha/year)	Estimated to Bacalar		Chance
		1999	2021	−5 ha/$
		7,474 ha	7,469 ha	
Provision				
Food	$364	$2,720,536	$2,718,716	−$1,820
Water	$8,618	$64,410,932	$64,367,842	−$43,090
Raw materials	$88	$657,712	$657,272	−$440
Subtotal		$67,789,180	$67,743,830	−$45,350
Regulation				
Climate regulation	$236	$1,763,864	$1,762,684	−$1,180
Moderation of extreme events	$8,077	$60,367,498	$60,327,113	−$40,385
Waste treatment	$2,189	$16,360,586	$16,349,641	−$10,945
Maintenance of soil fertility	$23	$171,902	$171,787	−$115
Biological control	$314	$2,346,836	$2,345,266	−$1,570
Subtotal		$81,010,686	$80,956,491	−$54,195
Support				
Maintenance of life cycles	$631	$4,716,094	$4,712,939	−$3,155
Subtotal		$4,716,094	$4,712,939	−$3,155
Cultural				
Aesthetic information	$1,214	$9,073,436	$9,067,366	−$6,070
Opportunities for recreation and tourism	$2,347	$17,541,478	$17,529,743	−$11,735
Existence, bequest values	$3,420	$25,561,080	$25,543,980	−$17,100
Inspiration for culture, art, and design	$2,672	$19,970,528	$19,957,168	−$13,360
Spiritual experience	$80	$597,920	$597,520	−$400
Information for cognitive development	$1,517	$11,338,058	$11,330,473	−$7,585
Subtotal		$84,082,500	$84,026,250	−$56,250
Total	$31,790	$237,598,460	$237,439,510	−$158,950

The most significant change was observed in inland wetlands, which decreased by 242.4 ha from 1999 to 2021. This reduction in area corresponds to a decline in the value of the ecosystem services they provide, amounting to 7,914,368 Int$/year (Fig. 4, Table 5). Regarding mangroves, they covered an area of 6,719.7 ha in 1999, with their ecosystem services valued at 523,662,720 Int$/year. However, the loss of 29 ha by 2021 resulted in a decrease in value of 2,337,780 Int$/year (Table 6). For lakes and rivers, the reduction of 5 ha from 1999 to 2021 corresponded to a decrease in the value of the ES provided by this ecosystem, amounting to 158,950 Int$/year (Table 7).

## Discussion

The capacity of an ecosystem to provide a service refers to its inherent potential, determined by the ecological and biophysical characteristics of the system, whereas effective provision pertains to the quantity of services generated and utilized by human communities (Haines-Young & Potschin, 2010). This distinction is particularly significant in ecosystems such as wetlands, which are dynamic and highly sensitive to both natural and anthropogenic disturbances (Bhowmik, 2019; Clarkson, Ausseil & Gerbeaux, 2013). This study aimed to assess changes in the potential capacity of the Bacalar Lagoon ecosystems to provide ecosystem services over time through spatial data analysis and the transfer of monetary values. While this approach offers a valuable tool for understanding general trends in service loss, it is important to emphasize that the results do not directly quantify the effective provision of ES.

Laguna Bacalar is a complex aquatic system where the interaction of biotic and abiotic elements, geomorphological processes, and climatic events has given rise to valuable ecosystems highly connected and sensitive to disturbances. This study observed that Laguna Bacalar, like many lagoon systems, is increasingly subject to anthropogenic pressures that drive land-use change and result in the loss of ES (Airoldi, Balata & Beck, 2008; Tomscha et al., 2019). Aligned with the general objective, changes in land cover classes were detected, reaffirming the effectiveness of remote sensing as a tool for assessing ecosystems conditions and their associated ecosystem services (Fernández-Díaz et al., 2022; Mendoza-González et al., 2012; Wong et al., 2015).

The spatiotemporal analysis of Laguna Bacalar, conducted from 1999 to 2021 using satellite imagery, revealed significant changes in land cover and land use, with 277 hectares of natural ecosystems converted into human-modified areas. This transformation is closely associated with the rapid physical expansion of urban areas near the coastline of the lagoon over the past two decades. Although Bacalar town historically experienced low to moderate population growth, other urban areas have nearly doubled in size over the last decade, with overall increases ranging from 100% to 300%, significantly intensifying pressure on the region’s natural ecosystems (Agua Clara, 2021). This pattern of variation aligns with observations in numerous ecosystems where anthropogenic activities have modified natural conditions (Aguilar-Medrano, 2023; Laurila-Pant et al., 2015; Seydewitz et al., 2023; Sutton et al., 2016). In this sense, the loss of diverse land cover represents modifications in the structure and function of ecosystems, leading to a decline in ES and, consequently, a negative impact on human well-being (Ellis, Romero Montero & Hernández Gómez, 2017; Long et al., 2022; Tolessa, Senbeta & Abebe, 2017).

In Bacalar, as in other regions of the world, the location and distribution of different wetland types are closely linked to the structure, functioning, and processes of each wetland type and, consequently, to the ES they provide (Hernández-Arana et al., 2015; Barbier, 2018; Waltham et al., 2020). The results indicate that the physiographic characteristics of Laguna Bacalar favor the establishment of human settlements and the development of anthropic activities along the west coast of the lagoon, while such activities are limited on the east coast. This pattern explains why the spatiotemporal variations of the ecosystems of Laguna Bacalar are concentrated on the west coast, primarily driven by the increasing demand for tourism services and infrastructure development (Checa Artasu, 2009; Giri et al., 2011; Secretary of Tourism of the Government of Mexico (SECTUR), 2014; Gómez Pech, Barrasa García & García de Fuentes, 2018; Espinosa-Sanchez, 2019). In contrast, the east coast’s flood zones restrict human activities (Hernández-Arana et al., 2015; Palafox-Juárez et al., 2022).

The intensification of human and tourism activities in recent years in the Bacalar region has highlighted its ecological fragility (Casarin et al., 2012; Gómez Pech, Barrasa García & García de Fuentes, 2018) and have raised growing concern about the sustainability of their ecosystem services and the implications for human well-being. Although the unit value transfer method may not fully capture local conditions or the ecological and socio-economic particularities of sites, we consider it a valuable strategy for promoting sustainable management of inland wetlands, mangroves, and Bacalar Lagoon. This method has been successfully applied to emphasize the importance of the environmental benefits provided by diverse ecosystems (Costanza et al., 2017); support the conservation of coral reefs (Ruiz de Gauna et al., 2021) and wetlands (Davidson et al., 2019); estimate the environmental costs of coastal ecosystems loss due to sea-level rise (Fernández-Díaz et al., 2022), and assess the impact of climate change on wetlands, among other applications.

The analysis of land cover changes and their relationship with the variation in the value of ES provided by Bacalar’s ecosystems revealed that, from 1999 to 2021, 277 ha of mangroves, inland wetlands, and the lake were modified. This modification led to an overall reduction in the value of the ES provided by these ecosystems, estimated at 10,411,098 Int$/year. The most significant changes were observed for inland wetlands, which decreased from 546.2 ha in 1999 to 303.8 ha in 2021, leading to a reduction in the value of their ecosystem services of approximately 7,914,368 Int$/year. This decline is of particular concern, as inland wetlands have a restricted distribution along the littoral margin and serve as the first line of defense for filtration and purification of organic matter inputs from upper watershed tributaries (Aguilar-Medrano, 2023; Barbier & Enchelmeyer, 2014; Davidson & Finlayson, 2019; Van Zanten et al., 2021). This degradation may increase the ecosystem’s vulnerability to anthropogenic impacts and climate change, while also impairing its supporting and regulating capacities. For example, Fernández-Díaz et al. (2022) report that the degradation of coastal ecosystems increases the risk of erosion and flooding, resulting in significant economic costs and heightened perception of vulnerability among human populations (Rulleau & Rey-Valette, 2017). Similarly, the degradation of inland wetlands has been reported to reduce habitat quality for wetland species and modify natural hydrological regimes (McCauley et al., 2015).

In the case of mangroves, the most prominent ecosystem in the study area, the modification of 29 ha may appear to be of low magnitude; however, given the increasing tourism promotion and intensification of anthropogenic activities in the region, it is crucial to highlight that further degradation of mangroves could significantly impact key ES vital for ecosystem health. These include regulation and climate services, water purification (Xu et al., 2018; Davidson & Finlayson, 2019) mitigation of extreme event, and erosion prevention (Koh et al., 2018; Waltham et al., 2020; Zhang, He & Yang, 2021). Additionally, the connectivity with neighboring ecosystems (Hernández-Arana et al., 2015) and the scenic beauty of the Bacalar system could be compromised, leading to adverse effects on the regional economy and human well-being in the medium and long term (Russi et al., 2013).

The analysis of the lakes and rivers ecosystems showed that between 1999 and 2021, 5 hectares were lost, corresponding to an estimated reduction in ES value of approximately 158,950 Int$/year. Bacalar Lagoon is a water body influenced by climatic seasonality and rainfall patterns (Carrillo et al., 2024; Hernández-Arana et al., 2015) which may experience changes in its surface area due to natural variation rather than actual losses (Xu et al., 2018). However, it is important to emphasize that the degradation of this ecosystem could have medium-and long-term consequences for the ES it provides, such as the availability of water resources and the health of adjacent ecosystems (Davidson & Finlayson, 2019), as well as impact on the structure and function of mangroves and wetlands. Furthermore, it could affect the provision of fundamental services for human health and well-being, such as water regulation, availability, and quality (Russi et al., 2013; Mitsch, Bernal & Hernandez, 2015; Grizzetti et al., 2016).

Bacalar’s ecosystems collectively provide a unique and aesthetic valuable landscape, contributing to numerous ecosystem services, most notably tourism and recreation. Over the past 20 years, its scenic beauty has become a resource that generates significant income for riverside communities (Secretary of Tourism of the Government of Mexico (SECTUR), 2014; Gómez Pech, Barrasa García & García de Fuentes, 2018; Sosa Ferreira & Martinez, 2016) and represents significant contributions to the GDP (INEGI, 2023). However, this economic reliance has driven exponential growth in infrastructure projects (Hirales-Cota et al., 2010), including real estate developments, road construction, material extraction sites, and, more recently, the Mayan Train. These activities have intensified pressure on Bacalar’s ecosystems, contributing to the deterioration and overloading of its wetlands and lagoon, ultimately affecting its scenic beauty and landscape. This degradation has resulted in total depreciation of Cultural ES valued at 4,127,542 Int$/year between 1999 and 2021, due to the modification of 277 hectares of mangroves, inland wetlands, and lakes and rivers.

The capacity of mangroves and wetlands to provide ecosystem services is directly linked to their extent, conservation status, and connectivity. However, the modification of 277 hectares represents a significant reduction in their potential to offer ES. Laguna Bacalar is a system where mangroves, the lagoon, and inland wetlands are intricately interconnected (Perry et al., 2009; Hernández-Arana et al., 2015; Pérez-Ceballos et al., 2021). An example of these interdependencies is the change in the lagoon’s color in 2021, caused by the inflow of terrigenous material into the water body following extraordinary rainfall associated with Tropical Storm Cristobal (Comisión Nacional del Agua (CONAGUA), 2020). This color change highlights the impact of losing 242 ha of inland wetlands, which previously acted as a protective barrier. The runoff of rainwater through deforested areas, in the absence of these inland wetlands, increased the direct input of materials into the lagoon, modifying its color and transparency for nearly two years, with direct implications for oxygen and nutrient concentrations (UN Environment, 2017; Álvarez-Legorreta, 2025), mortality of fauna such as the *Pomacea flagellata* snail (De Jesús-Navarrete et al., 2019; De Jesús-Navarrete, 2025), and a noticeable decline in the lagoon’s scenic beauty, negatively impacting the local and regional economy as well as the well-being of communities dependent on the lagoon’s ecosystem services.

The case of the Bacalar wetlands underscores the interconnected stages of the Ecosystem Services Cascade framework (Haines-Young & Potschin, 2010)—functions, capacity, provision, benefits, and values—and demonstrates how disruptions at the initial stages (functions and capacity) can cascade into diminished perceived benefits and values. Although our findings primarily highlight the potential capacity of these ecosystems, observations during extreme events revealed that effective provision is substantially limited in degraded systems.

Reversing this trend requires implementing targeted strategies to restore wetland capacity and enhance the effective provision of ecosystem services. These strategies should include the rehabilitation of degraded wetlands, the establishment of vegetative barriers in critical runoff areas, and the strict regulation of agricultural and industrial activities within the watershed. Furthermore, integrating community participation and adopting Nature-Based Solutions (O’Leary et al., 2023; Teutli Hernández et al., 2020) are essential for bolstering the resilience of wetlands against future extreme events. Such actions, combined with prioritizing the conservation of remaining wetlands, have the potential to restore a significant portion of the lost ecosystem service value, thereby enhancing ecological sustainability and improving human well-being in the region.

Finally, this study applied the unit value transfer method, enabling an approximation of the ecosystems’ potential capacity by assigning monetary values based on prior studies conducted in similar contexts. However, we acknowledge the limitations of this method, including its inability to fully account for critical contextual factors such as local ecological degradation, socioeconomic dynamics, and restrictions on access to and use of ecosystem services. Thus, it is imperative to complement such analyses with detailed local assessments to effectively optimize conservation and management strategies.

## Conclusions

Medium-resolution satellite imagery (Landsat 5 and Landsat 9) effectively assessed spatiotemporal changes in Laguna Bacalar’s ecosystem cover. It was also very helpful in valuing ecosystem services and in the environmental management of ecosystems in regions such as Bacalar.

The loss of 277 hectares of natural ecosystems between 1999 and 2021 has substantially diminished the capacity of wetlands to provide essential services, such as water regulation and purification. This underscores the urgent need for sustainable strategies that integrate conservation with tourism and urban development to mitigate further losses and ensure the long-term resilience of these ecosystems.

Our findings, derived from the unit value transfer method, highlight the monetary value of Bacalar’s wetlands in providing ecosystem services. While this approach is valuable, it has limitations, particularly in its inability to fully account for specific local dynamics or socioeconomic and ecological constraints. Complementing this method with primary assessments is essential to achieving more accurate and context-specific estimates.

Targeted assessments that incorporate local monitoring, community participation, and nature-based solutions are essential for enhancing the sustainability of these ecosystem services. These strategies should prioritize biodiversity conservation and the economic well-being of local communities, thereby promoting sustainable development. Highlighting the monetary value of these ecosystems can serve as a compelling message for decision-makers, reinforcing the need for their protection and responsible management.

## Additional Information and Declarations

### Data Availability

The following information was supplied regarding data availability:

All the information used to prepare this article is available at the Landsat sensor (https://earthexplorer.usgs.gov/).

## References

[ref-84] Agua Clara (2021). Laguna Bacalar Report Card. https://www.aguaclara-por-bacalar.org/_files/ugd/9a6bc4_324dfbf413cc4780a3ac4cb65aa16d94.pdf.

[ref-1] Aguilar-Medrano R (2023). Importancia de los humedales costeros de la península de Yucatán como centros de conexión ecológica para peces. Bioagrociencias.

[ref-2] Airoldi L, Balata D, Beck MW (2008). The gray zone: relationships between habitat loss and marine diversity and their applications in conservation. Journal of Experimental Marine Biology and Ecology.

[ref-3] Alpuche-Álvarez YA, Ochoa-Gaona S, Monzón-Alvarado CM, Cortina-Villar S (2019). Modernización agrícola y valoración sociocultural de los servicios ecosistémicos en paisajes mayas del sureste de México. Ecología Austral.

[ref-4] Álvarez-Legorreta T, Palafox-Juárez EB, Callejas-Jiménez ME (2025). Laguna de Bacalar. Señales de deterioro entre siete colores. Laguna de Bacalar. Equilibrio y Vulnerabilidad Entre Siete Colores.

[ref-5] Barbier EB (2016). The protective service of mangrove ecosystems: a review of valuation methods. Marine Pollution Bulletin.

[ref-6] Barbier EB (2018). The value of coastal wetland ecosystem services. Coastal Wetlands: An Integrated Ecosystem Approach.

[ref-7] Barbier EB, Enchelmeyer BS (2014). Valuing the storm surge protection service of US Gulf Coast wetlands. Journal of Environmental Economics and Policy.

[ref-8] Bateman IJ, Harwood AR, Mace GM, Watson RT, Abson DJ, Andrews B, Crowe A, Day BH, Dugdale S, Fezzi C, Foden J, Hadley D, Haines-Young R, Mark H, Kontoleon A, Lovett AA, Munday P, Pascual U, Paterson JS, Perino G, Sen A, Siriwardena G, van Soest D, Termansen M (2013). Bringing ecosystem services into economic decision making: land use in the UK. Science.

[ref-9] Bautista F, Palacio-Aponte G, Quintana P, Zinck JA (2011). Spatial distribution and development of soils in tropical karst areas from the Peninsula of Yucatan, Mexico. Geomorphology.

[ref-10] Bhowmik S (2019). Ecological and economic importance of wetlands and their vulnerability: a review. Current State and Future Impacts of Climate Change on Biodiversity.

[ref-11] Brander L (2013). Guidance manual on value transfer methods for ecosystem services.

[ref-12] Brander LM, de Groot R, Schägner JP, Guisado-Goñi V, van’t Hoff V, Solomonides S, McVittie A, Eppink F, Sposato M, Do L, Ghermandi A, Sinclair M, Thomas R (2024). Economic values for ecosystem services: a global synthesis and way forward. Ecosystem Services.

[ref-13] Burkhard B, Kroll F, Müller F, Windhorst W (2009). Landscapes’ capacities to provide ecosystem services—a concept for land-cover based assessments. Landscape Online.

[ref-14] Carrillo L, Yescas M, Nieto-Oropeza MO, Elías-Gutiérrez M, Alcérreca-Huerta JC, Palacios-Hernández E, Reyes-Mendoza OF (2024). Investigating the morphometry and hydrometeorological variability of a fragile tropical karstic lake of the Yucatán Peninsula: Bacalar Lagoon. Hydrology.

[ref-15] Casarin RS, Martinez GR, Mariño-Tapia I, Vanegas GP, Baldwin EM, Mancera EE (2012). Manmade vulnerability of the Cancun beach system: the case of hurricane wilma. CLEAN—Soil, Air, Water.

[ref-16] Checa Artasu M (2009). Apuntes sobre San Felipe de Bacalar: un fuerte militar español en el sur de Yucatán (1727–2009) (p. 11). UNESTRA PORTADA: Siglos XII al XV. Peones de las Mesnadas y Almogávares de la Corona de Aragón. Reproducción autorizada por la Real Academia de la Historia de la lámina 19 del álbum El Ejército y la Armada, de Manuel Giménez González, obra editada por el Servicio de Publicaciones del Estado Mayor del Ejército. https://martinchecaartasu.com/wp-content/uploads/2017/09/articulo-Apuntes-sobre-San-Felipe-de-Bacalar.pdf.

[ref-17] Chuvieco E, Huete A (2009). Fundamentals of satellite remote sensing.

[ref-18] Clarkson BR, Ausseil A-GE, Gerbeaux P (2013). Wetland ecosystem services. Ecosystem Services in New Zealand.

[ref-19] Comisión Nacional del Agua (CONAGUA) (2020). Reseña de la tormenta tropical “Cristobal” del Océano Atlántico, 1 al 10 de junio de 2020. https://smn.conagua.gob.mx/tools/DATA/Ciclones%20Tropicales/Ciclones/2020-Cristobal%20.pdf.

[ref-20] Comisión Nacional Forestal (CONAFOR) (2018). Análisis de los procesos de deforestación en Quintana Roo. https://kualicomunicacion.net/assets/CuadernilloCONAFOR.pdf.

[ref-21] Cortés-Gómez C, Cervantes-Martínez A, Arce-Ibarra AM (2023). Valoración sociocultural de los servicios ecosistémicos de la zona costera del Caribe mexicano. Economía Sociedad y Territorio.

[ref-22] Costanza R (2020). Valuing natural capital and ecosystem services toward the goals of efficiency, fairness, and sustainability. Ecosystem Services.

[ref-23] Costanza R, de Groot R, Sutton P, van der Ploeg S, Anderson SJ, Kubiszewski I, Farber S, Turner RK (2014). Changes in the global value of ecosystem services. Global Environmental Change.

[ref-24] Costanza R, de Groot R, Braat L, Kubiszewski I, Fioramonti L, Sutton P, Farber S, Grasso M (2017). Twenty years of ecosystem services: how far have we come and how far do we still need to go?. Ecosystem Services.

[ref-25] Davidson NC (2014). How much wetland has the world lost? Long-term and recent trends in global wetland area. Marine and Freshwater Research.

[ref-26] Davidson NC, Finlayson CM (2018). Extent, regional distribution and changes in area of different classes of wetland. Marine and Freshwater Research.

[ref-27] Davidson NC, Finlayson CM (2019). Updating global coastal wetland areas presented in Davidson and Finlayson (2018). Marine and Freshwater Research.

[ref-28] Davidson NC, Van Dam AA, Finlayson CM, McInnes RJ (2019). Worth of wetlands: revised global monetary values of coastal and inland wetland ecosystem services. Marine and Freshwater Research.

[ref-29] de Groot R, Brander L, van der Ploeg S, Costanza R, Bernard F, Braat L, Christie M, Crossman N, Ghermandi A, Hein L, Hussain S, Kumar P, McVittie A, Portela R, Rodriguez LC, ten Brink P, van Beukering P (2012). Global estimates of the value of ecosystems and their services in monetary units. Ecosystem Services.

[ref-30] De Jesús-Navarrete A, Palafox-Juárez EB, Callejas-Jiménez ME (2025). Caracol chivita: ¿es preocupante su cambio poblacional en la Laguna de Bacalar?. Laguna de Bacalar. Equilibrio y Vulnerabilidad Entre Siete Colores.

[ref-31] De Jesús-Navarrete A, Ocaña-Borrego FA, Oliva-Rivera JJ, De Jesús-Carrillo RM, Vargas-Espositos AA (2019). Abundance, distribution, and secondary production of the apple snail Pomacea flagellata (Say, 1829) in Bacalar Lake, a tropical karstic system in southern Mexico. Studies on Neotropical Fauna and Environment.

[ref-32] Ellis EA, Navarro Martínez A, García Ortega M, Hernández Gómez IU, Chacón Castillo D (2020). Forest cover dynamics in the Selva Maya of Central and Southern Quintana Roo, Mexico: deforestation or degradation?. Journal of Land Use Science.

[ref-33] Ellis EA, Romero Montero JA, Hernández Gómez IU (2017). Deforestation processes in the state of Quintana Roo, Mexico: the role of land use and community forestry. Tropical Conservation Science.

[ref-34] Espinosa Coria H (2013). El origen del proyecto turístico Cancún, México. Una valoración de sus objetivos iniciales a 42 años de su nacimiento. LiminaR Estudios Sociales y Humanísticos.

[ref-35] Espinosa-Sanchez J (2019). Quintana Roo y Bacalar en el siglo XXI. http://192.100.164.85/handle/20.500.12249/1473.

[ref-36] Fernández-Díaz VZ, Canul Turriza RA, Kuc Castilla A, Hinojosa-Huerta O (2022). Loss of coastal ecosystem services in Mexico: an approach to economic valuation in the face of sea level rise. Frontiers in Marine Science.

[ref-37] Fu WJ, Jiang PK, Zhou GM, Zhao KL (2014). Using Moran’s I and GIS to study the spatial pattern of forest litter carbon density in a subtropical region of southeastern China. Biogeosciences.

[ref-38] Giri C, Ochieng E, Tieszen LL, Zhu Z, Singh A, Loveland T, Masek J, Duke N (2011). Status and distribution of mangrove forests of the world using earth observation satellite data. Global Ecology and Biogeography.

[ref-39] Goldenberg R, Kalantari Z, Cvetkovic V, Mörtberg U, Deal B, Destouni G (2017). Distinction, quantification and mapping of potential and realized supply-demand of flow-dependent ecosystem services. The Science of the Total Environment.

[ref-40] Gómez Pech EH, Barrasa García S, García de Fuentes A, Alvarado-Sizzo L, López L (2018). El Pueblo Mágico de Bacalar: construcción de un imaginario y ocupación del suelo en el litoral de la Laguna de los Siete Colores, Quintana Roo, México. Turismo, Patrimonio y Representaciones Espaciales.

[ref-41] Gopal B (2013). Future of wetlands in tropical and subtropical Asia, especially in the face of climate change. Aquatic Sciences.

[ref-42] Grizzetti B, Lanzanova D, Liquete C, Reynaud A, Cardoso AC (2016). Assessing water ecosystem services for water resource management. Environmental Science and Policy.

[ref-43] Haines-Young R, Potschin M (2010). The links between biodiversity, ecosystem services and human well-being. Ecosystem Ecology: A New Synthesis.

[ref-44] Harris Geospatial Solutions (2016). Envi (Version 5.4). https://www.l3harris.com/all-capabilities/geospatial-data-imagery.

[ref-45] Hernández-Arana H, Vega-Zepeda A, Ruíz-Zárate MA, Falcón-Álvarez L, López – Adame H, Herrera-Silveira J, Kaster J, Islebe GA, Calme S, Leon-Cortes JL (2015). Transverse coastal corridor: from freshwater lakes to coral reefs ecosystems. Biodiversity and Conservation of the Yucatan Peninsula.

[ref-47] Hirales-Cota M, Espinoza-Avalos J, Schmook B, Ruiz-Luna A, Ramos-Reyes R (2010). Drivers of mangrove deforestation in Mahahual-Xcalak, Quintana Roo, southeast Mexico. Ciencias Marinas.

[ref-48] Huchin Ochoa SA, Navarro-Martínez A, Ellis EA, Hernández Gómez IU (2022). Deforestación en el municipio de Bacalar, Quintana Roo, México durante el período 1993–2017. Madera y Bosques.

[ref-49] INEGI (2010). Red hidrográfica. Escala 1:50 000. Edición 2.0. Subcuenca hidrográfica RH33Ac R. Bahía de Chetumal. Cuenca Bahía de Chetumal y Otras. RH Yucatán Este (Quintana Roo). https://www.inegi.org.mx/app/biblioteca/ficha.html?upc=889463129868.

[ref-50] INEGI (2023). Producto interno bruto por entidad federativa (PIBE). https://www.inegi.org.mx/contenidos/saladeprensa/boletines/2023/PIBEF/PIBEF2022.pdf.

[ref-51] Johnston RJ, Wainger LA (2015). Benefit transfer for ecosystem service valuation: an introduction to theory and methods.

[ref-52] Koh HL, Teh SY, Kh’Ng XY, Raja Barizan RS (2018). Mangrove forests: protection against and resilience to coastal disturbances. Journal of Tropical Forest Science.

[ref-53] Kovács-Hostyánszki A, Espíndola A, Vanbergen AJ, Settele J, Kremen C, Dicks LV (2017). Ecological intensification to mitigate impacts of conventional intensive land use on pollinators and pollination. Ecology letters.

[ref-54] Laurila-Pant M, Lehikoinen A, Uusitalo L, Venesjärvi R (2015). How to value biodiversity in environmental management?. Ecological Indicators.

[ref-55] Lomnicky GA, Herlihy AT, Kaufmann PR (2019). Quantifying the extent of human disturbance activities and anthropogenic stressors in wetlands across the conterminous United States: results from the national wetland condition assessment. Environmental Monitoring and Assessment.

[ref-56] Long X, Lin H, An X, Chen S, Qi S, Zhang M (2022). Evaluation and analysis of ecosystem service value based on land use/cover change in Dongting Lake wetland. Ecological Indicators.

[ref-57] May-Arias EM, Arroyo Arcos L, Hernández Aguilar ML (2024). Servicios ecosistémicos culturales de los cenotes del municipio de Tulum, Quintana Roo: usos, beneficios y amenazas. Investigaciones Geográficas.

[ref-58] McCauley LA, Anteau MJ, van der Burg MP, Wiltermuth MT (2015). Land use and wetland drainage affect water levels and dynamics of remaining wetlands. Ecosphere.

[ref-59] MEA (2005). Millennium ecosystem assessment. Ecosystems and human well-being: biodiversity synthesis.

[ref-60] Mendoza-González G, Martínez ML, Lithgow D, Pérez-Maqueo O, Simonin P (2012). Land use change and its effects on the value of ecosystem services along the coast of the Gulf of Mexico. Ecological Economics.

[ref-61] Miranda F, Beltrán E (1958). Estudios acerca de la vegetación. Los Recursos Naturales del Sureste y su Aprovechamiento. Instituto Nacional de Recursos Naturales Renovables, A.C. México, D.F. Tomo II.

[ref-62] Mitsch WJ, Bernal B, Hernandez ME (2015). Ecosystem services of wetlands. International Journal of Biodiversity Science, Ecosystem Services and Management.

[ref-63] O’Leary BC, Fonseca C, Cornet CC, de Vries MB, Degia AK, Failler P, Furlan E, Garrabou J, Gil A, Hawkins JP, Krause-Jensen D, Le Roux X, Peck MA, Pérez G, Queirós AM, Różyński G, Sanchez-Arcilla A, Simide R, Sousa Pinto I, Trégarot E, Roberts CM (2023). Embracing nature-based Solutions to promote resilient marine and coastal ecosystems. Nature-Based Solutions.

[ref-64] Olofsson P, Foody GM, Herold M, Stehman SV, Woodcock CE, Wulder MA (2014). Good practices for estimating area and assessing the accuracy of land change. Remote Sensing of Environment.

[ref-65] Palafox-Juárez EB, Herrera-Silveira J, Teutli-Hernández C, Callejas-Jiménez M, Torrescano-Valle N (2022). Mangroves and Wetlands of Bacalar, Quintana Roo, Mexico, dataset. Mendeley Data. https://data.mendeley.com/datasets/vrg7x9kvt5/3.

[ref-66] Pérez-Ceballos R, Canul-Macario C, Pacheco-Castro R, Pacheco-Ávila J, Euán-Ávila J, Merino-Ibarra M (2021). Regional hydrogeochemical evolution of groundwater in the ring of cenotes, Yucatán (Mexico): an inverse modelling approach. Water.

[ref-67] Perry E, Paytan A, Pedersen B, Velazquez-Oliman G (2009). Groundwater geochemistry of the Yucatan Peninsula, Mexico: constraints on stratigraphy and hydrogeology. Journal of Hydrology.

[ref-68] Plummer ML (2009). Assessing benefit transfer for the valuation of ecosystem services. Frontiers in Ecology and the Environment.

[ref-200] Porto RG, De Almeida RF, Cruz-Neto O, Tabarelli M, Viana BF, Peres CA, Lopes AV (2020). Pollination ecosystem services: a comprehensive review of economic values, research funding and policy actions. Food Security.

[ref-69] R Core Team (2021). R: a language and environment for statistical computing.

[ref-70] Rebolledo-Vieyra M, Ávalos JE, Islebe GA, Arana HAH (2009). Aspectos geológicos de la cuenca del Caribe. El Sistema Ecológico de la Bahía de Chetumal/Corozal: Costa Occidental del Mar Caribe.

[ref-71] Richardson L, Loomis J, Kroeger T, Casey F (2015). The role of benefit transfer in ecosystem service valuation. Ecological Economics.

[ref-72] Ruiz de Gauna I, Markandya A, Greno F, Warman J, Arce N, Navarrete A, Rivera M, Kobelkowsky R, Vargas M, Hernandez M (2021). Economic valuation of the ecosystem services of the Mesoamerican Reef, and the allocation and distribution of these values. https://www.econstor.eu/handle/10419/237493.

[ref-73] Rulleau B, Rey-Valette H (2017). Forward planning to maintain the attractiveness of coastal areas: choosing between seawalls and managed retreat. Environmental Science & Policy.

[ref-74] Russi D, ten Brink P, Farmer A, Bandura T, Coates D, Dorster J, Kumar R, Davidson N (2013). The economics of ecosystems and biodiversity for water and wetlands: a final consultation draft.

[ref-75] Sánchez-Quinto A, Costa JC, Zamboni NS, Sanches FHC, Principe SC, Viotto EV, Casagranda E, da Veiga-Lima FA, Possamai B, Faroni-Perez L (2020). Development of a conceptual framework for the management of biodiversity and ecosystem services in the Mexican Caribbean. Biota Neotropica.

[ref-76] Sannigrahi S, Chakraborti S, Joshi PK, Keesstra S, Sen S, Paul SK, Kreuter U, Sutton PC, Jha S, Dang KB (2019). Ecosystem service value assessment of a natural reserve region for strengthening protection and conservation. Journal of Environmental Management.

[ref-77] Secretary of Tourism of the Government of Mexico (SECTUR) (2005). El turismo de segundas residencias en México. Secretaría de Turismo. https://cedocvirtual.sectur.gob.mx/janium/Documentos/000776Pri0000.pdf.

[ref-78] Secretary of Tourism of the Government of Mexico (SECTUR) (2014). Bacalar, Quintana Roo. Pueblos Mágicos. Características y atractivos. Secretaría de Turismo. https://www.gob.mx/sectur/es/articulos/bacalar-quintana-roo.

[ref-79] Seydewitz T, Pradhan P, Landholm DM, Kropp JP (2023). Deforestation drivers across the tropics and their impacts on carbon stocks and ecosystem services. Anthropocene Science.

[ref-80] Shepard CC, Crain CM, Beck MW (2011). The protective role of coastal marshes: a systematic review and meta-analysis. PLOS ONE.

[ref-81] Sosa Ferreira AP, Martinez CI (2016). El turismo de cruceros y la transformación del paisaje: Majahual. El Periplo Sustentable.

[ref-82] Sutton PC, Anderson SJ, Costanza R, Kubiszewski I (2016). The ecological economics of land degradation: impacts on ecosystem service values. Ecological Economics.

[ref-83] Swangjang K, Panishkan K (2021). Assessment of factors that influence carbon storage: an important ecosystem service provided by mangrove forests. Heliyon.

[ref-85] Taylor S, Kumar L, Reid N (2011). Accuracy comparison of Quickbird, Landsat TM and SPOT 5 imagery for Lantana camara mapping. Journal of Spatial Science.

[ref-86] Teutli Hernández C, Herrera-Silveira JA, Cisneros-de la Cruz DJ, Roman-Cuesta RM (2020). Guía para la restauración ecológica de manglares: lecciones aprendidas.

[ref-87] Teutli-Hernández C, Palafox-Juárez EEB, Cisneros de la Cruz D, Esquivel JJL, Guerra-Cano L, Robles-Toral PJ, Herrera Silveira JA, Rosas-Luis R, Castr-Pérez JJ (2024). Restauración de ecosistemas costeros Quintana Roo, México. Elementos de Ecología de la Zona Marino Costera del Mar Caribe Mexicano.

[ref-88] The Economics of Ecosystems & Biodiversity (2010). Mainstreaming the economics of nature: a synsthesis of the approach, conclusions and recommendations of TEEB. Environment.

[ref-89] Tolessa T, Senbeta F, Abebe T (2017). Land use/land cover analysis and ecosystem services valuation in the central highlands of Ethiopia. Forests Trees and Livelihoods.

[ref-90] Tomscha S, Deslippe J, de Róiste M, Hartley S, Jackson B (2019). Uncovering the ecosystem service legacies of wetland loss using high-resolution models. Ecosphere.

[ref-91] Torrescano-Valle N, Palafox-Juárez EB, Álvarez-Legorreta T, Cedeño-Vázquez R, Corona-Figueroa MF, Palafox-Juárez EB, Callejas-Jiménez MEELB (2025). Bacalar desde la mirada de El Colegio de la Frontera Sur. Laguna de Bacalar. Equilibrio y Vulnerabilidad Entre Siete Colores.

[ref-92] Troy A, Wilson MA (2006). Mapping ecosystem services: practical challenges and opportunities in linking GIS and value transfer. Ecological Economics.

[ref-93] UN Environment (2017). A framework for freshwater ecosystem management. https://www.unep.org/resources/publication/framework-freshwater-ecosystem-management.

[ref-94] Uribe-Martínez A, Berriel-Bueno D, Chávez V, Cuevas E, Almeida KL, Fontes JVH, van Tussenbroek BI, Mariño-Tapia I, Liceaga-Correa MÁ, Ojeda E, Castañeda-Ramírez DG, Silva R (2022). Multiscale distribution patterns of pelagic rafts of sargasso (Sargassum spp.) in the Mexican Caribbean (2014–2020). Frontiers in Marine Science.

[ref-95] Vanbergen AJ, Insect Pollinators Initiative (2013). Threats to an ecosystem service: pressures on pollinators. Frontiers in Ecology and the Environment.

[ref-96] Van Zanten BT, Brander LM, Gutierrez Torres D, Uyttendaele GYP, Herrera Garcia LD, Patrama D, Kaczan DJ (2021). The economics of large-scale Mangrove conservation and restoration in Indonesia.

[ref-97] Velázquez-Salazar S, Rodríguez-Zúñiga MT, Alcántara-Maya JA, Villeda-Chávez E, Valderrama-Landeros L, Troche-Souza C, Vázquez-Balderas B, Pérez-Espinosa I, Cruz-López MI, Ressl R, De la Borbolla DVG, Paz O, Aguilar-Sierra V, Hruby F, Muñoa-Coutiño JH (2021). Manglares de México. Actualización y análisis de los datos 2020. https://www.biodiversidad.gob.mx/publicaciones/librosDig/pdf/Manglares2013_Indice.pdf.

[ref-98] Waltham NJ, Elliott M, Lee SY, Lovelock C, Duarte CM, Buelow C, Simenstad C, Nagelkerken I, Claassens L, Wen CKC, Barletta M, Connolly RM, Gillies C, Mitsch WJ, Ogburn MB, Purandare J, Possingham H, Sheaves M (2020). UN decade on ecosystem restoration 2021–2030—what chance for success in restoring coastal ecosystems?. Frontiers in Marine Science.

[ref-99] Wong CP, Jiang B, Kinzig AP, Lee KN, Ouyang Z (2015). Linking ecosystem characteristics to final ecosystem services for public policy. Ecology Letters.

[ref-100] Xu X, Jiang B, Tan Y, Costanza R, Yang G (2018). Lake-wetland ecosystem services modeling and valuation: progress, gaps and future directions. Ecosystem Services.

[ref-101] Yan J, Zhu J, Zhao S, Su F (2023). Coastal wetland degradation and ecosystem service value change in the Yellow River Delta, China. Global Ecology and Conservation.

[ref-102] Yanez-Montalvo A, Gómez-Acata S, Águila B, Hernández-Arana H, Falcón LI (2020). The microbiome of modern microbialites in Bacalar Lagoon, Mexico. PLOS ONE.

[ref-103] Zhang X, He S, Yang Y (2021). Evaluation of wetland ecosystem services value of the Yellow River Delta. Environmental Monitoring and Assessment.

